# Metabolic Basis for Thyroid Hormone Liver Preconditioning: Upregulation of AMP-Activated Protein Kinase Signaling

**DOI:** 10.1100/2012/475675

**Published:** 2012-07-31

**Authors:** Luis A. Videla, Virginia Fernández, Pamela Cornejo, Romina Vargas

**Affiliations:** ^1^Molecular and Clinical Pharmacology Program, Institute of Biomedical Sciences, Faculty of Medicine, University of Chile, Santiago, Chile; ^2^Faculty of Medicine, Diego Portales University, Santiago, Chile

## Abstract

The liver is a major organ responsible for most functions of cellular metabolism and a mediator between dietary and endogenous sources of energy for extrahepatic tissues. In this context, adenosine-monophosphate- (AMP-) activated protein kinase (AMPK) constitutes an intrahepatic energy sensor regulating physiological energy dynamics by limiting anabolism and stimulating catabolism, thus increasing ATP availability. This is achieved by mechanisms involving direct allosteric activation and reversible phosphorylation of AMPK, in response to signals such as energy status, serum insulin/glucagon ratio, nutritional stresses, pharmacological and natural compounds, and oxidative stress status. Reactive oxygen species (ROS) lead to cellular AMPK activation and downstream signaling under several experimental conditions. Thyroid hormone (L-3,3′,5-triiodothyronine, T_3_) administration, a condition that enhances liver ROS generation, triggers the redox upregulation of cytoprotective proteins affording preconditioning against ischemia-reperfusion (IR) liver injury. Data discussed in this work suggest that T_3_-induced liver activation of AMPK may be of importance in the promotion of metabolic processes favouring energy supply for the induction and operation of preconditioning mechanisms. These include antioxidant, antiapoptotic, and anti-inflammatory mechanisms, repair or resynthesis of altered biomolecules, induction of the homeostatic acute-phase response, and stimulation of liver cell proliferation, which are required to cope with the damaging processes set in by IR.

## 1. Introduction

In mammals, the liver is a major organ responsible for metabolic functions including most of the pathways for intermediary catabolism, glucose, lipoprotein, and plasma protein biosynthesis, biotransformation of xenobiotics, excretion, and secretion of different metabolites and mediators [1]. The liver functions as a mediator between dietary and endogenous sources of energy and extrahepatic organs that continuously require energy, mainly the brain and erythrocytes, under cycling conditions between fed and fasted states. In the fed state, where insulin action predominates, digestion-derived glucose is converted to pyruvate via glycolysis, which is oxidized to produce energy, whereas fatty acid oxidation is suppressed. Excess glucose can be either stored as hepatic glycogen or channelled into *de novo* lipogenesis. In the fasted state, considerable liver fuel metabolism changes occur due to decreased serum insulin/glucagon ratio, with higher glucose production as a consequence of stimulated glycogenolysis and gluconeogenesis (from alanine, lactate, and glycerol). Major enhancement in fatty acid oxidation also occurs to provide energy for liver processes and ketogenesis to supply metabolic fuels for extrahepatic tissues [2]. For these reasons, the liver is considered as the metabolic processing organ of the body, and alterations in liver functioning affect whole-body metabolism and energy homeostasis. Moreover, understanding the signaling mechanisms regulating liver energy metabolism is crucial for the management of metabolic diseases or for developing preconditioning strategies aimed at preventing organ injury [1].

 In this context, adenosine-monophosphate- (AMP-) activated protein kinase (AMPK) is the downstream component of a protein kinase cascade acting as an intracellular energy sensor regulating physiological energy dynamics by limiting anabolic pathways, to prevent excessive adenosine triphosphate (ATP) utilization, and by stimulating catabolic processes, to increase ATP production [3]. Thus, the understanding of the mechanisms by which liver AMPK coordinates hepatic energy metabolism represents a crucial point of convergence of regulatory signals monitoring systemic and cellular energy status [3–5].

## 2. Liver AMPK: Structure and Regulation

**Figure 1:**
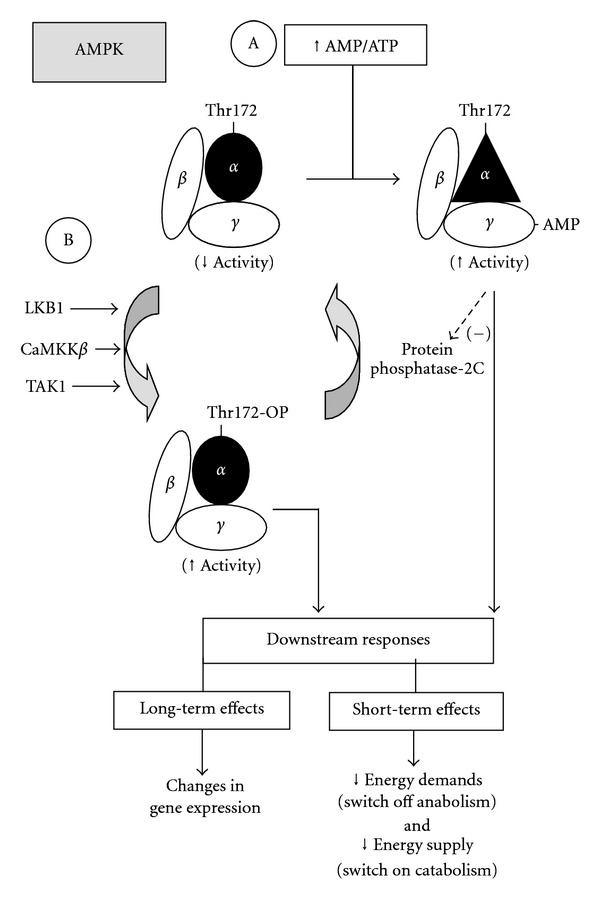
Regulation of AMP-activated protein kinase (AMPK) by (A) direct allosteric activation and (B) reversible phosphorylation and downstream responses maintaining intracellular energy balance. Abbreviations: LKB1, tumor suppressor LKB1 kinase; CaMKK*β*, Ca^2+^-calmodulin-dependent kinase kinase-*β*; TAK1, transforming growth-factor-*β*-activated kinase-1.

 AMPK, a serine/threonine kinase, is a heterotrimeric complex comprising a catalytic subunit *α* and two regulatory subunits *β* and *γ* involved in heterotrimer formation and ligand sensing (Figure 1). The *α* subunit has a threonine residue (Thr172) within the activation loop of the kinase domain, with the C-terminal region being required for association with *β* and *γ* subunits. The *β* subunit associates with *α* and *γ* by means of its C-terminal region [6], whereas the *γ* subunit has four cystathionine *β*-synthase (CBS) motifs, which bind AMP or ATP in a competitive manner [7].

 Regulation of liver AMPK activity involves both direct allosteric activation and reversible phosphorylation. AMPK is allosterically activated by AMP through binding to the regulatory subunit-*γ*, which induces a conformational change in the kinase domain of subunit *α* that protects AMPK from dephosphorylation of Thr172 [8], probably by protein phosphatase-2C [9] (Figure 1(A)). Activation of AMPK requires phosphorylation of Thr172 in its *α* subunit, which can be attained by either (i) tumor suppressor LKB1 kinase following enhancement in the AMP/ATP ratio [10], a kinase that plays a crucial role in AMPK-dependent control of liver glucose and lipid metabolism [11]; (ii) Ca^2+^-calmodulin-dependent protein kinase kinase-*β* (CaMKK*β*) that phosphorylates AMPK in an AMP-independent, Ca^2+^-dependent manner [12]; (iii) transforming growth-factor-*β*-activated kinase-1 (TAK1) [13] (Figure 1(B)), an important kinase in hepatic Toll-like receptor 4 signaling in response to lipopolysaccharide [14]. Among these kinases, the relevance of CaMKK*β* and TAK1 in liver AMPK activation remains to be established in metabolic stress conditions. Both allosteric and phosphorylation mechanisms are able to elicit over 1000-fold increase in AMPK activity [15], thus allowing the liver to respond to small changes in energy status in a highly sensitive fashion (Figure 1).

Liver AMPK is activated in response to different metabolic stresses, including those that increase ATP utilization (activation of biosynthetic pathways) or that reduce ATP production (hypoxia, glucose deprivation, inhibition of mitochondrial oxidative phosphorylation) [16]. A clear example of liver AMPK modulation occurs in the transition from fasted to fed state, which implies physiological changes in energy dynamics. During refeeding, liver AMPK*α*1 activity is decreased within 1 h, an effect that is exacerbated for both AMPK*α*1 and AMPK*α*2 isoforms in the 1 to 24 h period [17]. These changes are compatible with the increase in plasma insulin, reported to reduce hepatic AMPK activity, and diminution in glucagon, shown to activate hepatic AMPK. Besides, liver AMPK can be regulated by ghrelin, glucocorticoids, and the adipokines resistin and adiponectin, in addition to pharmacological and natural drugs including polyphenols, 5-aminoimidazole-4-carboxamide-1-*β*-D-ribofuranoside (AICAR), and the antidiabetic drugs metformin and thiazolidinediones [2].

In addition to rapid AMPK regulation through allosterism and reversible phosphorylation, long-term effects of AMPK activation induce changes in hepatic gene expression. This was demonstrated for (i) the transcription factor carbohydrate-response element-binding protein (ChREBP), whose Ser568 phosphorylation by activated AMPK blocks its DNA binding capacity and glucose-induced gene transcription under hyperlipidemic conditions [18]; (ii) liver sterol regulatory element-binding protein-1c (SREBP-1c), whose mRNA and protein expression and those of its target gene for fatty acid synthase (FAS) are reduced by metformin-induced AMPK activation, decreasing lipogenesis and increasing fatty acid oxidation due to malonyl-CoA depletion [19]; (iii) transcriptional coactivator transducer of regulated CREB activity-2 (TORC2), a crucial component of the hepatic gluconeogenic program, was reported to be phosphorylated by activated AMPK. This modification leads to subsequent cytoplasmatic sequestration of TORC2 and inhibition of gluconeogenic gene expression [20], a mechanism underlying the plasma glucose-lowering effects of adiponectin and metformin through AMPK activation by upstream LKB1 [21]. Interestingly, the polyphenol epigallocatechin-3-gallate- (EGCG-) induced AMPK-dependent repression of gluconeogenic genes is mediated by an LKB1-independent pathway, relying on CaMKK*β* activation through generation of reactive oxygen species (ROS) [22].

 In conclusion, activation of AMPK in the liver is a key regulatory mechanism controlling glucose and lipid metabolism, inhibiting anabolic processes, and enhancing catabolic pathways in response to different signals, including energy status, serum insulin/glucagon ratio, nutritional stresses, pharmacological and natural compounds, and oxidative stress status.

## 3. Reactive Oxygen Species (ROS) and AMPK Activation

The high energy demands required to cope with all the metabolic functions of the liver outlined in the INTRODUCTION are met by fatty acid oxidation under conditions of both normal blood glucose levels and hypoglycemia, whereas glucose oxidation is favoured in hyperglycemic states, with consequent generation of ROS [1]. Due to the electronic structure of oxygen in the ground state, one-electron transfer reactions occur, leading to the generation of ROS such as superoxide radical (O_2_^•−^), hydrogen peroxide (H_2_O_2_), and hydroxyl radical (HO^•^). In addition, secondary ROS are produced, including alkoxyl (RO^•^) and peroxyl (ROO^•^) radicals or hydroperoxides (ROOH) derived from biomolecules (R), hypochlorous acid (HClO), and the electronically exited state singlet oxygen (O_2_*) [23, 24]. At the cellular level, ROS lead to a wide spectrum of responses depending (i) on the cell type, with different cells differing in the basal antioxidant status, (ii) the level of ROS achieved, and (iii) the duration of the exposure [23, 24]. Under normal conditions, ROS occur at relatively low levels due to their fast processing by antioxidant mechanisms, whereas at acute or prolonged high ROS levels, severe oxidation of biomolecules and dysregulation of signal transduction and gene expression is achieved, with consequent cell death through necrotic and/or apoptotic-signaling pathways. On the other hand, transient and moderate ROS generation may trigger signals regulating either protein function, through reversible oxidation or nitrosation of protein sulfhydryls, and/or gene expression, via modulation of the activity of specific protein kinases, protein phosphatases, or redox-sensitive transcription factors, with induction of cytoprotective responses [23–25]. 

AMPK is activated by several physiological and pathological conditions that are characterized by concomitant ROS generation, such as heat shock in isolated hepatocytes [26], hypoxia/ischemia in heart muscle [27], exercise in skeletal muscle [28], and ATP depletion through mitochondrial electron flow inhibition by antimycin A or azide [29]. However, direct regulation of AMPK by ROS was first reported by Choi et al. [30] using H_2_O_2_ (25 to 600 *μ*M) in NIH-3T_3_ cells, in association with increased AMP/ATP ratios and AMPK*α*1-Thr172 phosphorylation. Although these events were blocked by pretreatment with the potent free-radical scavenger dimethyl sulfoxide [30], the nature of the upstream kinase(s) activating AMPK was not established, although 1 mM H_2_O_2_ AMPK stimulation via CaMKK*β* activation in fibroblasts from LKB1^−/−^ mice has been reported [31]. In addition, AMPK activation is observed in different cell-cultures exposed to 10 *μ*M to 1 mM H_2_O_2_ [32] showing N-acetylcysteine (NAC) sensitivity [33], or under conditions of low glucose concentrations triggering mitochondrial ROS production [34]. Interestingly, AMPK activation under hypoxic conditions was proposed to be induced by enhancement in the AMP/ATP ratio [16]; however, recent data demonstrate that as cellular O_2_ levels decrease, mitochondrial complex III acts as an O_2_-sensor by releasing ROS into the intermembrane space, which upon diffusion into the cytosol trigger AMPK activation [35].

 Phytochemicals have been shown to be involved in regulation of a variety of metabolic processes, with therapeutic effects for obesity, diabetes, and cardiovascular diseases, including green tea EGCG, red pepper capsaicin, and soybean genistein [22, 36], polyphenols that generate ROS related to free-radical-induced chain reactions [37]. Due to this feature, activation of the AMPK signaling pathway is achieved by genistein, EGCG, and capsaicin as an NAC-sensitive response [36, 38]. However, in these studies the upstream kinase(s) involved in AMPK phosphorylation was not established. Furthermore, mouse hepatocytes subjected to EGCG exhibit increased ROS generation in the presence of NADPH, leading to AMPK phosphorylation through upstream CaMKK*β* and suppression of hepatic gluconeogenesis [22]. Hepatocyte AMPK activation by EGCG is suppressed by a cell membrane permeable catalase that eliminates H_2_O_2_; however, the mechanism of CaMKK*β* activation by ROS remains to be determined. In this model system, EGCG did not induce phosphorylation of LKB1, an alternate candidate kinase for AMPK activation [22].

 In conclusion, ROS trigger cellular AMPK activation under different experimental conditions, including (i) direct *in vitro* H_2_O_2_ addition to cell cultures, (ii) *in vitro* and* in vivo* conditions underlying ROS generation, or (iii) ROS production coupled to free-radical chain reactions induced by polyphenols, with responses being time and concentration dependent.

## 4. Thyroid Hormone (L-3,3′,5-Triiodothyronine, T_3_), Metabolic Regulation, and ROS Production

 T_3_ is important for the normal function of most mammalian tissues, with major actions on O_2_ consumption and metabolic rate, thus determining enhancement in fuel consumption for oxidation processes and ATP repletion [1]. T_3_ acts predominantly through nuclear receptors (TR) *α* and *β*, forming functional complexes with retinoic X receptor that bind to thyroid hormone response elements (TRE) to activate gene expression [39]. T_3_ calorigenesis is primarily due to the induction of enzymes related to mitochondrial electron transport and ATP synthesis, catabolism, and some anabolic processes via upregulation of genomic mechanisms [40]. The net result of this T_3_ action is the enhancement in the rate of O_2_ consumption of target tissues such as liver, which may be contributed by secondary processes induced by T_3_ such as (i) energy expenditure due to higher active cation transport, (ii) energy loss due to futile cycles coupled to increase in catabolic and anabolic pathways [41], and (iii) O_2_ equivalents used in hepatic ROS generation both in hepatocytes and Kupffer cells [40]. In addition, T_3_-induced higher rates of mitochondrial oxidative phosphorylation are likely to induce higher levels of ATP, which are partially balanced by intrinsic uncoupling afforded by induction of uncoupling proteins by T_3_ [42]. In agreement with this view, the cytosolic ATP/ADP ratio is decreased in hyperthyroid tissues, due to simultaneous stimulation of ATP synthesis and consumption [43].

Although T_3_ influences most pathways of intermediary metabolism, actions on lipid metabolism are particularly relevant. In this respect, T_3_ accelerates TG turnover and chylomicron clearance rate [44], with fatty acids derived from adipose tissue lipolysis being the primary source of substrate for T_3_-induced calorigenesis via *β*-oxidation, and whereas the early increase in lipogenesis serves simply to maintain fat stores [45]. Thus, the concentration and turnover of free fatty acids are increased in hyperthyroidism, resulting from a T_3_-induced increase in (i) lipolysis, as the result of higher adipose tissue sensitivity to lipolytic hormones, and (ii) fatty acid *β*-oxidation to CO_2_ as well as to ketone bodies [44]. Regulation of fatty acid oxidation is mainly attained by carnitine palmitoyltransferase I*α* (CPT-I*α*), catalyzing the transport of fatty acids from cytosol to mitochondria for *β*-oxidation, and acyl-CoA oxidase (ACO), catalyzing the first rate-limiting reaction of peroxisomal *β*-oxidation, enzymes that are induced by both T_3_ and peroxisome proliferator-activated receptor *α* (PPAR-*α*) [46, 47]. Furthermore, PPAR-*α*-mediated upregulation of CPT-I*α* mRNA is enhanced by PPAR-*γ* coactivator 1*α* (PGC-1*α*), which in turn augments T_3_ induction of CPT-I*α* expression [48]. Interestingly, PGC-1*α* is induced by T_3_ [49], AMPK activation [33], and ROS [50], thus establishing potential links between T_3_ action, ROS generation, and AMPK activation with the onset of mitochondrial biogenesis and fatty acid *β*-oxidation.

**Figure 2:**
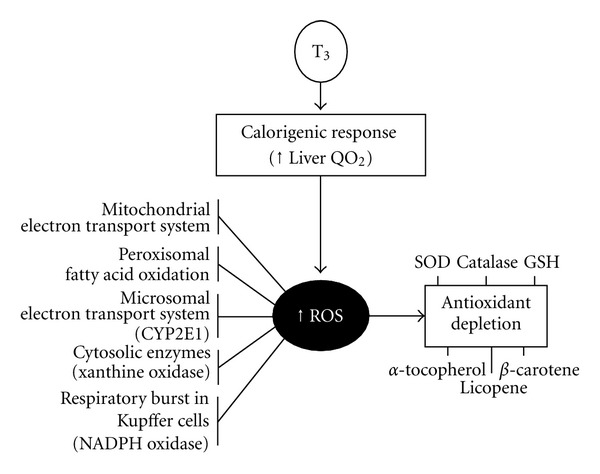
Calorigenic response of thyroid hormone (T_3_) and its relationship with O_2_ consumption, reactive oxygen species (ROS) generation, and antioxidant depletion in the liver. Abbreviations: CYP2E1, cytochrome P450 isoform 2E1; GSH, reduced glutathione; QO_2_, rate of O_2_ consumption; SOD, superoxide dismutase.

**Figure 3:**
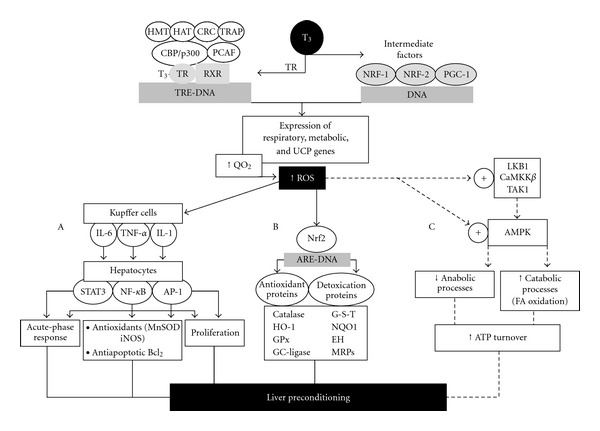
Genomic signaling mechanisms in T_3_ calorigenesis and liver reactive oxygen species (ROS) production leading to (A) upregulation of cytokine expression in Kupffer cells and hepatocyte activation of genes conferring cytoprotection, (B) Nrf2 activation controling expression of antioxidant and detoxication proteins, and (C) activation of the AMPK cascade regulating metabolic functions. Abbreviations: AP-1, activating protein 1; ARE, antioxidant responsive element; CaMKK*β*, Ca^2+^-calmodulin-dependent kinase kinase-*β*; CBP, CREB binding protein; CRC, chromatin remodelling complex; EH, epoxide hydrolase; HO-1, hemoxygenase-1; GC-Ligase, glutamate cysteine ligase; GPx, glutathione peroxidase; G-S-T, glutathione-S-transferase; HAT, histone acetyltransferase; HMT, histone arginine methyltransferase; IL-1 (6), interleukin 1 (6); iNOS, inducible nitric oxide synthase; LKB1, tumor suppressor LKB1 kinase; MnSOD, manganese superoxide dismutase; MRPs, multidrug resistance proteins; NF-*κ*B, nuclear factor-*κ*B; NQO1, NADPH-quinone oxidoreductase-1; NRF-1 (2), nuclear respiratory factor-1 (2); Nrf2, nuclear receptor-E2-related factor 2; PCAF, p300/CBP-associated factor; RXR, retinoic acid receptor; PGC-1, peroxisome proliferator-activated receptor-*γ* coactivator-1; QO_2_, rate of O_2_ consumption; STAT3, signal transducer and activator of transcription 3; TAK1, transforming-growth-factor-*β*-activated kinase-1; TNF-*α*, tumor necrosis factor-*α*; TR, T_3_ receptor; TRAP, T_3_-receptor-associated protein; TRE, T_3_ responsive element; UCP, uncoupling proteins; (—), reported mechanisms; (- - - -), proposed mechanisms.

Enhancement in cellular O_2_ consumption by T_3_ increases ROS production at several subcellular sites of hepatocytes and in the respiratory burst of Kupffer cells (Figure 2) [40]. Liver ROS generation leads to activation of the transcription factors nuclear factor-*κ*B (NF-*κ*B), activating protein 1 (AP-1), and signal transducer and activator of transcription 3 (STAT3) at the Kupffer cell level, with upregulation of cytokine expression (TNF-*α*, IL-1, IL-6), which upon interaction with specific receptors in hepatocytes trigger the expression of cytoprotective proteins (Figure 3(A)). These responses and the promotion of hepatocyte and Kupffer-cell proliferation represent hormetic effects reestablishing redox homeostasis, promoting cell survival, and protecting the liver against ischemia-reperfusion injury [51]. T_3_ liver preconditioning also involves the activation of the Nrf2-Keap1 defense pathway [52, 53], upregulating antioxidant proteins, phase-2 detoxifying enzymes, and multidrug resistance proteins, members of the ATP binding cassette (ABC) superfamily of transporters (Figure 3(B)) [24, 54]. In agreement with T_3_-induced liver preconditioning, T_3_ or L-thyroxin afford preconditioning against IR injury in the heart, in association with activation of protein kinase C [55] and attenuation of p38 and c-Jun-N-terminal kinase activation [56], and in the kidney, in association with heme oxygenase-1 upregulation [57].

In conclusion, T_3_ is a key metabolic regulator coordinating short-term and long-term energy needs [39], with major actions on liver metabolism. These include promotion of (i) gluconeogenesis and hepatic glucose production, and (ii) fatty acid oxidation coupled to enhanced adipose tissue lipolysis, with higher fatty acid flux to the liver and consequent ROS production (Figure 2) and redox upregulation of cytoprotective proteins affording liver preconditioning (Figure 3).

## 5. Thyroid Hormone and AMPK Activation: Skeletal Muscle and Heart

The influence of T_3_ administration on cellular AMPK signaling pathway has been studied in skeletal muscle and heart, as a response to physiological energy needs during performance.

In skeletal muscle, T_3_ increases the levels of numerous proteins involved in glucose uptake (GLUT4), glycolysis (enolase, pyruvate kinase, triose phosphate isomerase), fatty acid oxidation (carnitine palmitoyl transferase-1, mitochondrial thioesterase I), and uncoupling protein-3, effects that are achieved through enhanced transcription of TRE-containing genes (Figure 3) [58]. Apart from these genomic actions, T_3_ also exerts rapid nongenomic *in vitro *and *in vivo* effects involving AMPK and Akt/PKB, thus mediating uptake and oxidation of both glucose and fatty acids [59]. In studies under different experimental conditions, T_3_ was reported to upregulate the expression of muscle AMPK [60, 61], leading to AMPK activation by phosphorylation [60–64], with concomitant phosphorylation of the AMPK target protein acetyl-CoA carboxylase (ACC) [60, 63, 64]. Skeletal muscle AMPK activation is characterized by (i) being a rapid and transient response [62, 63], (ii) upstream activation by Ca^2+^-induced mobilization and CaMKK*β* activation [64], (iii) upstream upregulation of LKB1 expression, which requires association with STRAD and MO25 for optimal phosphorylation/activation of AMPK [61, 65], and (iv) stimulation of mitochondrial fatty acid *β*-oxidation [60, 64]. In addition to these effects, T_3_-induced muscle AMPK activation was found to trigger two major downstream signaling pathways, namely, (i) peroxisome proliferator-activated receptor-*γ* coactivator-1*α* (PGC-1*α*) mRNA expression [63] and phosphorylation [61], a transcriptional regulator for genes related to mitochondrial biogenesis, fatty acid oxidation, and gluconeogenesis [66] and (ii) cyclic AMP response element binding protein (CREB) phosphorylation [61], which in turn induces PGC-1*α* expression in liver tissue [67], thus reinforcing mechanism (i). These data indicate that AMPK phosphorylation of PGC-1*α* initiates many of the important gene regulatory functions of AMPK in skeletal muscle [68].

In heart, hyperthyroidism increased glycolysis and sarcolemmal GLUT4 levels by the combined effects of AMPK activation and insulin stimulation, with concomitant increase in fatty acid oxidation that is proportional to enhanced cardiac mass and contractile function [69].

## 6. Thyroid Hormone, AMPK Activation, and Liver Preconditioning

**Figure 4:**
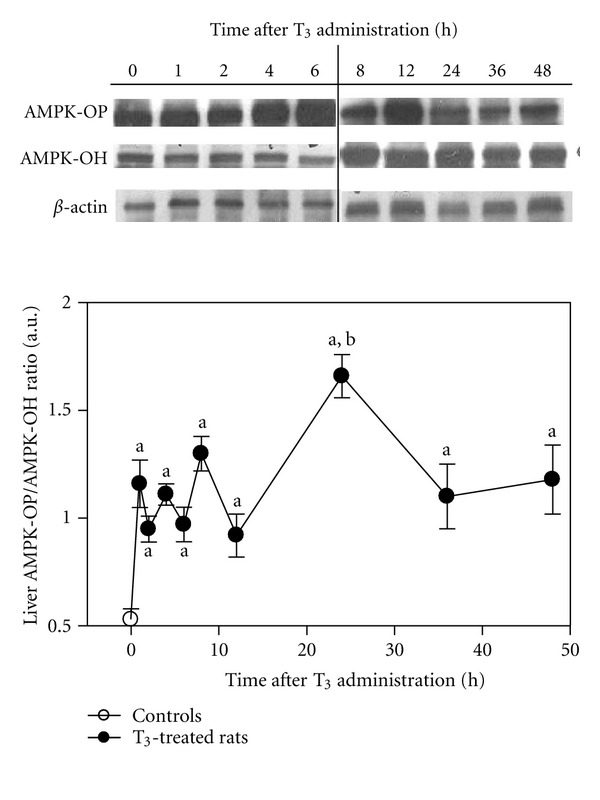
Effect of L-3,3′,5-triiodothyronine (T_3_) administration on rat liver AMP-activated protein kinase (AMPK). Male Sprague-Dawley rats (Animal facility of the Institute of Biomedical Sciences, Faculty of Medicine, University of Chile) weighing 180–200 g were housed on a 12-hour light/dark cycle and were provided with rat chow and water *ad libitum*. Animals received a single intraperitoneal dose of 0.1 mg of T_3_/kg body weight or equivalent volumes of hormone vehicle (0.1 N NaOH, controls shown at time zero). Liver samples (100–500 mg) frozen in liquid nitrogen were homogenized and suspended in a buffer solution pH 7.9 containing 10 mM HEPES, 1 mM EDTA, 0.6% Nonidet P-40, 150 mM NaCl, and protease inhibitors (1 mM phenylmethylsulfonyl fluoride, 1 *μ*g/mL aprotinin, 1 *μ*g/mL leupeptin, and 1 mM orthovanadate). Soluble protein fractions (50 *μ*g) were separated on 12% polyacrylamide gels using SDS-PAGE [85] and transferred to nitrocellulose membranes [86], which were blocked for 1 hour at room temperature with TBS-containing 5% bovine serum albumin. The blots were washed with TBS containing 0.1% Tween 20 and hybridized with either rabbit polyclonal antibody for phospho-AMPK (62 kDa) and mouse monoclonal antibodies for AMPK (62 kDa) (Abcam, Inc., Cambrige, MA) or *β*-actin (43 kDa) (ICN Biomedicals, Inc., Aurora, OH). In all determinations, anti-*β*-actin was used as internal control for cytosolic fractions. After extensive washing, the antigen-antibody complexes were detected using horseradish peroxidase goat anti-rabbit IgG or goat anti-mouse IgG and a SuperSignal West Pico Chemiluminescence kit detection system (Pierce, Rockford, IL). Values shown correspond to the means ± SEM for 3 to 4 separate animals. ^a^ *P* < 0.05 compared to control values at time zero; ^b^ *P* < 0.05 compared to T_3_-treated rats at 1, 2, 4, 6, 8, 12, 36, and 48 h, assessed by one-way ANOVA and the Newman-Keuls, test.

Recent studies by our group revealed that administration of a single dose of 0.1 mg T_3_/kg to rats activates liver AMPK (Figure 4; unpublished work). Western blot analysis of hepatic AMPK showed enhancement in phosphorylated AMPK/nonphosphorylated AMPK ratios in T_3_-treated rats over control values, an effect that is significant in the time period of 1 to 48 hours after hormone treatment (Figure 4). Administration of a substantially higher dose (0.4 mg T_3_/kg) resulted in decreased liver AMPK activation at 4 h to return to control values at 6 h after treatment [63], a dose that may induce adverse effects in the liver associated with the thyrotoxicosis state developed [70].

 Activation of liver AMPK by T_3_ may be of relevance in terms of promotion of fatty acid oxidation for ATP supply, supporting hepatoprotection against IR injury (Figure 3(C)). This proposal is based on the high energy demands underlying effective liver preconditioning for full operation of hepatic antioxidant, antiapoptotic, and anti-inflammatory mechanisms [71, 72], oxidized biomolecules repair or resynthesis [24, 40], induction of the homeostatic acute-phase response [73], and promotion of hepatocyte [74] and Kupffer cell [75] proliferation, mechanisms that are needed to cope with the damaging processes set in by IR [76–78]. T_3_ liver preconditioning [51, 74, 79], in addition to that afforded by n-3 long-chain polyunsaturated fatty acids given alone [80, 81] or combined with T_3_ at lower dosages [82], or by iron supplementation [83], constitutes protective strategies against hepatic IR injury that may have clinical application in human liver transplantation and hepatic resections. This is a most important issue considering that pharmacological approaches, gene therapy, and strategies underlying chemically induced moderate oxidative stress development have not reached the clinical setting due to toxicity problems, side effects, or difficulties in implementation, with the exception of ischemic preconditioning that remains controversial at present time [80, 84]. For these reasons, studies on the molecular mechanisms underlying T_3_-induced liver AMPK activation (Figure 4) are currently under assessment in our laboratory, information that precedes the future design of protocols assessing T_3_ effects in IR-related injury in human liver surgery.
